# Simulation of a Novel Tubular Microalgae Photobioreactor with Aerated Tangent Inner Tubes: Improvements in Mixing Performance and Flashing-Light Effects

**DOI:** 10.1155/2020/8815263

**Published:** 2020-07-10

**Authors:** Xuyang Cui, Junhong Yang, Yuanzheng Feng, Wenwen Zhang

**Affiliations:** ^1^Key Laboratory of Efficient Utilization of Low and Medium Grade Energy, MOE, Tianjin University, Tianjin 300350, China; ^2^School of Mechanical Engineering, Tianjin University, Tianjin 300350, China

## Abstract

At present, large-scale and high-efficiency microalgal cultivation is the key to realizing the technology for carbon capture and storage (CCS) and bioresource recovery. Meanwhile, tubular photobioreactors (PBRs) have great potential for microalgal cultivation due to their high productivity. To improve the mixing performance and flashing-light effect, a novel tube PBR with the inner tube tangential to the outer tube was developed, whose radial aeration pores are situated along the length of the inner tube. The direction of aeration, aeration rate, light/dark cycle period (L/D), light-time ratio, average turbulent kinetic energy (TKE), and degree of synergy between the velocity and direction of the light field in the PBR were optimized by a computational fluid dynamics (CFD) simulation and field synergy theory. The results show that a downwards aeration direction of 30° and an aeration rate of 0.7 vvm are the most conducive to reducing the dead zone and improving the light/dark cycle frequency. Compared to the concentric double-tube PBR, the light/dark cycle frequency and light time of the tangent double-tube PBR increased by 78.2% and 36.2% to 1.8 Hz and 47.8%, respectively, and the TKE was enhanced by 48.1% from 54 to 80 cm^2^·s^−2^. Meanwhile, field synergy theory can be extended and applied to the design of tubular microalgae PBRs, and the average synergy of the light and velocity gradients across the cross-section increased by 38% to 0.69. The tangential inner tube aeration structure generated symmetrical vertical vortices between the light and dark areas in the PBR, which significantly improved the mixing performance and flashing-light effect. This novel design can provide a more suitable microenvironment for microalgal cultivation and is promising for bioresource recovery applications and improving the yield of microalgae.

## 1. Introduction

Microalgae are considered to be one of the most promising technologies for carbon capture and storage (CCS) and have been identified as a superior feedstock for biodiesel production [1, 2]. Microalgae are also important biological resources for recycling. However, the low biomass productivity of microalgal cultivation systems is a bottleneck in commercial production, which leads to the high cost of microalgae biofuels [3]. Before this situation can be reversed, a significant improvement in volumetric productivity is required [4]. This goal will require improved microalgae genetic engineering and microalgae process engineering [5]. However, compared to other engineering processes, it is difficult to achieve breakthroughs in genetic engineering. Therefore, it is very important to improve the cultivation system related to mass and radiation energy transfer, nutrient absorption, and growth rate [6]. Focusing on high efficiency and large-scale microalgal cultivation, tubular photobioreactors (PBRs) are considered one of the most suitable culture systems. However, their weak mass transfer, wall growth, photoinhibition, and photolimitations have limited their development [7]. Several tube-based photobioreactors (PBRs) have been designed for microalgal cultivation, but the experimental characterization of the flow field in PBR is difficult and costly [8]. The development of computational fluid dynamics (CFD) has facilitated the study of hydrodynamic performance and the structural design of tubular PBRs [1].

Tubular PBRs have great potential for microalgal cultivation due to their high productivity compared with open ponds [9]. CFD has been widely used in flow field simulations of tubular PBR to optimize the mixing of the culture medium. Azizi et al. [10] studied a new type of tubular PBR with an embedded static mixer via CFD simulation and showed that the mixing conditions and fluid dynamics in the proposed PBR were better than those in the traditional PBR. Later, Qin and Wu [11] studied tubular PBR with a 4-unit Kenics mixer and a 1-unit Kenics mixer using CFD, thereby confirming two methods for increasing the efficiency of the light/dark cycle frequency. Gómez-Pérez et al. [12] studied the effect of wall turbulence promoters (that is, the profile of the inner tube PBR wall) and found that the wall turbulence promoters offer better mixing behavior than standard PBRs at flow velocities of 0.2–0.25 m/s, although the energy uptake is 60–80% lower. Based on this study, Lei et al. [13] developed a new type of tubular PBR with spiral ribs, which has good mixing performance and flashing-light effects. In addition, Wu et al. [14] studied the flow dynamics of a spiral tube by CFD and showed stable Dean vortex motion along the axial coordinates, although there was no vortex in the tubular section. The mixing performance of a spiral tube PBR is much better than that of a tubular PBR. Perner-Nochta and Posten [15] subsequently focused on a tubular PBR using a static mixer for spiral tube PBRs, applying CFD and trajectory analysis to examine the scale-down/scale-up effects. Gómez-Pérez et al. [16] discussed four types of twisted tubular PBRs by CFD simulation and provided an important contribution towards constructing an efficient tubular PBR. Meanwhile, Ye et al. [8] simulated a novel type of continuous lantern-shaped PBR (LDT) that can generate a vortex flow field and enhance the mass transfer in a microalgae solution, similar to the effect of twisting a tubular PBR.

In addition, Su et al. [17] studied PBRs; they designed a destabilized mixing bar using CFD, and the results show that the vertical speed along the light path caused by the mixing bar helped achieve homogenous mixing of the culture medium, thereby improving the photosynthetic efficiency of the algal cells. Pruvost et al. [18] designed a ring-shaped PBR that can accurately control the light and provide very effective mixing, especially along the light gradient of the culture. Pruvost et al. [19] proposed an attenuated ring-shaped vortex flow caused by the tangential inlet, which increased the displacement of the microalgae along the light gradient. Similarly, Sato et al. [20] invented three novel closed PBRs that used aeration to mix, including a parabola, a pipe, and a diamond. The simulation results show that the parabola has advantages in mass transfer and that the pipe is the best performer for *Chaetoceros calcitrans* cultivation (1.8 times higher performance than that of the control dome). Therefore, mixing along the light gradient can improve the efficiency of photosynthesis, which is the main principle of new PBR designs. Moreover, Cheng et al. [1] proposed using a tangential jet that generates a large clockwise vertical vortex and several secondary vortices in a novel jet-charged tangential swirl plate PBR (JTSP); this method can significantly reduce the dead zone and increase the light/dark circle frequency. According to a report by Guo et al. [21], in 1998, the field synergy principle was proposed to improve the convective heat transfer rate by analyzing the boundary layer heat transfer mechanism. The effects depended on the synergy level of the velocity and the thermal flow field, as well as the synergy angle between the velocity and the thermal gradient [22]. Under the same speed and temperature boundary conditions, the higher the level of synergy is, the higher the heat transfer intensity [23]. Based on the similarities between heat and mass transfer, Chen et al. [24] derived mathematical synergy equations for mass transfer and used them to optimize the mass transfer of a photocatalytic oxidation reactor. In general, light and mixing are very important for microalgal cultivation. Mixing along the direction of light is conducive to the transmission of light but also to the mixing of the gas phase (CO_2_), liquid phase (culture medium), and solid phase (microalgae cells), thereby producing a suitable flow environment and flashing-light effect, which are beneficial to microalgal cultivation [1]. However, few works have reported the synergy between the light field and flow field of the tubular PBR used for microalgal cultivation.

In this work, we propose a novel tangent double tube with radial aeration pores along the length of the inner tube, in which the inner tube is tangential to the outer tube in order to strengthen the synergy between the light and velocity gradients. The direction of aeration, the aeration rate, the light/dark cycle period (L/D), the light-time ratio, the average turbulent kinetic energy (TKE), and the degree of synergy between the velocity and the direction of the light field in the PBR were analyzed by computational fluid dynamics (CFD) and the field synergy principle.

## 2. Design and Numerical Models of a Novel PBR

### 2.1. Development of a Novel PBR

**Figure 1:**
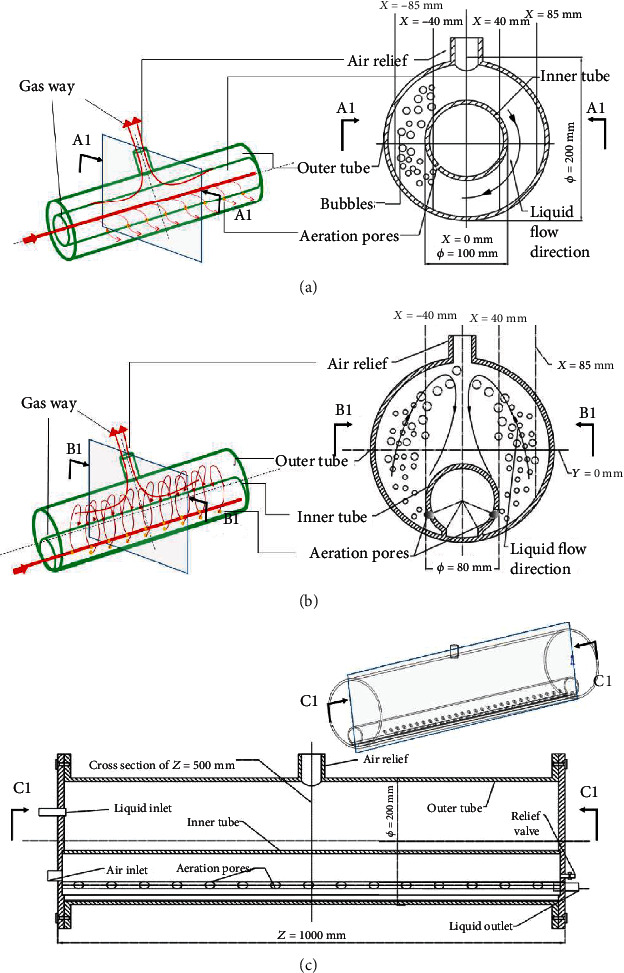
Conceptual structures for a novel double-tube photobioreactor (PBR). (a) The concentric double-tube PBR in our previous work; (b, c) the novel tangent double-tube PBR in this work.

In our previous study [25], we illustrated the initial concept of a novel double-tube PBR (see Figure 1(a)), which included a concentric double tube with aeration pores along the tube length. Previous research results indicate that the concentric double-tube PBR proposed in this previous work can help facilitate efficient microalgal cultivation. As shown in Figure 1(a), microalgae are cultivated in the annular space between the outer tube and the inner tube and are aerated through a series of radial pores installed along the length of the inner tube, which can provide air and mix the cultures.

As shown in Figures 1(b) and 1(c), the new tangent double-tube PBR in this work can reduce the volume of the dark areas, thereby increasing the proportion of the light and dark areas. Fernández et al. [26] discussed the light distribution of the tube and found that under normal outdoor conditions, the tube had almost no dark areas and a diameter of 0.06 m. Provided the zone that has a light path less than 60 mm is a light zone, the light/dark ratio for the tangent with an outer tube of 200 mm diameter and an inner tube of 80 mm diameter is 0.648 and increases by 31.4% compared to 0.493 for a tube of the same size. To some extent, increasing the proportion of light and dark areas is beneficial for microalgal cultivation. Barbosa et al. [27] studied the effects of cycle time (10–100 s) and light fraction (0.1–1) on the growth rate and biomass yield of *Dunaliella tertiolecta* in airlift reactors, and the results show that an increase in the light fraction, for a constant medium cycle time, led to an increase in the biomass yield and growth rate. In addition, compared with the vortex attenuation flow caused by coaxial inlets [28], this new aeration method is expected to reduce the attenuation of the vortex flow along the length of the tube, thereby obtaining better performance.

As shown in Figure 1(b), a 60° arc was removed at the bottom of the inner tube, and the inner and outer tubes were connected by a glass panel to alleviate algae stuck to the wall near the corner. As shown in Figure 1, the origin is located at the axis of the outer tube, the *x*-axis direction extends from the origin to the top of the outer tube (illuminated area), and the *y*-axis direction extends from the origin to the right side of the outer tube. As presented by Sato et al. [20], the parabola reactor (modified from a conventional panel by removing the corners) had an acute angle to alleviate the algae stuck to the wall near the corner. Aeration resulted in the two symmetric rows of pores located on the inner tube producing a parabolic flow, which resulted in high mixing efficiency of the algae, nutrients, and dissolved gas. Similarly, the simulation results of Sato et al. [20] showed that algae, nutrients, and dissolved gases mix best in a parabola. Notably, the parabola proposed by Sato et al. is different from the structure shown in Figure 1(a), and it is not an annular volume. In addition, the cultivation effect of microalgae in the annular space ventilated by the outer circular tube is the best [29], likely because of the annular space of the circular tube and the radial aeration caused by a series of pores. Therefore, these tangential double tubes seem to be better in microalgal cultivation.

### 2.2. Theoretical Model and CFD Simulation

#### 2.2.1. The Synergy Principle for Microalgal Cultivation

The synergy between the velocity of the fluid particles in the laminar flow field and the temperature gradient can be calculated using Equation (1). If the direction of the fluid velocity is closer to the direction of the heat flow, the effect of convective heat transfer in the laminar flow will be better [24, 30]:

(1) $$\begin{array}{c} \alpha =\arccos\left[\frac{\left(\vec{U}∙\nabla T\right)}{\left(\left|\vec{U}\right|∙\left|\nabla T\right|\right)}\right]. \end{array}$$

Considering that the heat flux in the PBR is almost constant, and the direction of the light under the artificial light source is also constant, the synergy between the light field and the flow field can be calculated as Equation (2), where *I* is the illumination intensity and $\vec{U}$ is the flow velocity. The angle between the velocity field and the light direction indicates the degree of synergy. When the angle *α* is 90°, the degree of coordination is the worst. When *α* is 180° or 0°, the degree of synergy is optimal. Here, the absolute value of ∣cos*α*∣ is used to analyze the synergy. The ∣cos*α*∣ value is 1, so the degree of synergy is the best.

(2) $$\begin{array}{c} \left|\cos\alpha \right|=\left|\frac{\left(\vec{\text{gard}}I\vec{∙U}\right)}{\left(\left|\vec{U}\right|∙\left|\vec{\text{gard}}I\right|\right)}\right|. \end{array}$$

#### 2.2.2. Numerical Mathematics Models

The Eulerian–Eulerian two-flow model and the standard *k*‐*ε* turbulence model were used to simulate the hydrodynamic characteristics of the flow in the novel PBRs. The slurry of the freshwater green algae *C. vulgaris* displayed Newtonian behavior for biomass concentrations from 0.5 to 60 kg·m^−3^, with an increase in viscosity from 1 to 1.6 m·s^−1^. The slurry of *Nannochloris* sp. and *Phaeodactylum tricornutum* gave similar results, which are also similar to the physical properties of water. Thus, a two-phase flow of air-water was adopted during the simulation [31]. Moreover, the UDF was used to analyze the ∣cos*α*∣ curve.

*(1) Euler–Euler Two-Phase Model*. The continuity equation was calculated as

(3) $$\begin{array}{c} \frac{\partial }{\partial t}\left(r_{q}\rho_{q}\right)+\nabla \left(r_{q}\rho_{q}\overset{⃑}{U_{q}}\right)=0\;\left(q=1,g\right), \end{array}$$

where *t*, *r*_*q*_, *ρ*_*q*_, and $\vec{U_{q}}$ are the time, volume fraction, density, and velocity vector of phase *q*, respectively.

The momentum equation is provided in

(4) $$\begin{array}{c} \frac{\partial }{\partial t}\left(r_{q}\rho_{q}\overset{⃑}{U_{q}}\right)+\nabla \left[r_{q}\left(\rho_{q}\overset{⃑}{U_{q}}\overset{⃑}{U_{q}}\right)\right]=-r_{q}\nabla p_{q}+r_{q}\rho_{q}\left(\overset{⃑}{F_{q}}+\overset{⃑}{{\text{F}}_{lift,q}}\right)+\nabla \left[r_{q}\mu_{q}\left(\nabla \overset{⃑}{U_{q}}+{\left(\nabla \overset{⃑}{U_{q}}\right)}^{T}\right)\right]\;\left(q=1,g\right), \end{array}$$

where *p*_*q*_, $\overset{⃑}{F_{q}}$, $\overset{⃑}{F_{lift,q}}$, and *μ*_*q*_ are the partial pressure, body force, lift force, and dynamic coefficient of the viscosity of phase *q*, respectively. The superscript *T* indicates the turbulent term [32]. Microalgae growth needs constant temperature, so this project could be simplified to the mass transfer of a two-phase flow without heat transfer.

*(2) Turbulence Model*. The turbulence in the continuous phase (nutrient) is captured using the standard *k*‐*ε* model. The standard *k*‐*ε* equation was summarized from the experimental phenomena based on the semiempirical transport equation of the turbulence energy (*k*) and turbulent dissipation rate (*ε*) [31].

The transport equation of the turbulence energy was calculated using

(5) $$\begin{array}{c} \frac{\partial }{\partial t}\left(r_{q}\rho_{q}k_{q}\right)+\nabla \left[r_{q}\rho_{q}k_{q}\vec{U_{q}}\right]=\nabla \left(\frac{r_{q}\mu_{q,t}}{\sigma_{k}}\cdot \nabla k_{q}\right)+r_{q}G_{k,q}+\underset{k,g}{\prod }r_{q}\rho_{q}-r_{q}\rho_{q}\varepsilon_{q}. \end{array}$$

Next, the transport equation of the turbulent dissipation rate was calculated via

(6) $$\begin{array}{c} \frac{\partial }{\partial t}\left(r_{q}\rho_{q}\varepsilon_{q}\right)+\nabla \left[r_{q}\rho_{q}\varepsilon_{q}\vec{U_{q}}\right]=\nabla \left(\frac{r_{q}\mu_{q,t}}{\sigma_{\varepsilon }}\cdot \nabla \varepsilon_{q}\right)+\frac{r_{q}\varepsilon_{q}}{k_{q}}\left(C_{1\varepsilon }G_{k,q}\right)-\frac{C_{2\varepsilon }r_{q}\rho_{q}\varepsilon_{q}^{2}}{k_{q}}+\underset{\varepsilon ,g}{\prod }r_{q}\rho_{q}. \end{array}$$

The turbulence kinetic energy, *G*_*k*,*q*_, results from the average velocity gradient. *П*_*k*,*q*_ and *П*_*ε*,*q*_ describe the impact of the dispersed phase on the continuous phase. *μ*_*q*,*t*_ is the turbulence viscosity coefficient, and *μ*_*q*,*t*_ was calculated using

(7) $$\begin{array}{c} \mu_{q,t}=\frac{C_{\mu }\rho_{q}k_{q}^{2}}{\varepsilon_{q}}, \end{array}$$

where *C*_1*ε*_, *C*_2*ε*_, and *C*_*μ*_ are the model constants fixed at 1.44, 1.92, and 0.09, respectively, and the turbulent Prandtl numbers *σ*_*k*_ and *σ*_*ε*_ were set as 1.0 and 1.3, respectively.

#### 2.2.3. Initial Boundary Conditions of Simulation

The inlet conditions were set based on the gas-liquid two-phase velocity inlet. The aeration pores in the tangent structure were located on the two sides, while the pores in the concentric structure were placed on only one side and canted downwards by 45° (*x* < 0). The aeration rates were set to 0.3, 0.7, and 1 vvm, and these PBRs were initially filled with water. The outlet conditions were set based on the pressure-outlet boundary, and the walls were all set without slip boundary conditions. Meanwhile, the angle of light incidence was simplified as 90°.

The calculation step length was 0.1 s to conduct unsteady calculations, and the convergence residual was 10^−4^. The three-dimensional meshes of the novel PBRs were designed with the ANSYS ICEM CFD 12.1 software (64 bits). Due to the cylinder structures in the tangent double tube, the computational domain was dispersed through the unstructured grid. The grids of the tangent double tube were 340 thousand, while the grids of the concentric double tube were 870 thousand. The grid independence experiment showed that when the grid number was greater than the previous grid number, the difference in the calculation results was not obvious. Four positions of *x* = ±40 mm and *x* = ±85 mm were chosen to monitor and analyze the data. The data of ∣cos*α*∣ were calculated via UDF (User-Defined Function) to assess the extent of the synergy between the light direction and the velocity vector.

#### 2.2.4. Calculation of the Light/Dark Cycle Period

During this simulation, spherical particles are used to represent the algal cells with a number (*n*) of 20, a diameter (*d*) of 10 *μ*m, and a density (*ρ*) of 1000 kg·m^−3^, and their positions are recorded every 0.1 s. The maximum particle tracking time is set to 60 s. The discrete random walk model is used to simulate the motion of the particles in the PBRs [8]. The particles are affected by the drag force (*F*_*D*_) and pressure gradient force (*F*_*P*_), which are shown in [33]

(8) $$\begin{array}{c} F_{D}=\frac{1}{8}\pi \rho_{d}d^{2}C_{D}\left|{\vec{v}}_{f}-{\vec{v}}_{d}\right|\left({\vec{v}}_{f}-{\vec{v}}_{d}\right), \end{array}$$

(9) $$\begin{array}{c} F_{P}=\frac{\pi d^{3}{\rho_{d}}_{f}}{6}\frac{d{\vec{v}}_{f}}{dt}, \end{array}$$

where ${\vec{v}}_{f}$ is the velocity vector and ${\vec{v}}_{d}$ is the particle velocity vector. Subscripts *p* and *f* denoted the particle and the fluid, respectively. The value of *C*_*D*_ is 0.44, which is the drag coefficient required to compute the drag force.

According to the previous experimental work [25], the light zone of the double-tube PBR is 0.04 < *y* < 50 mm, and the dark zone is −50 mm < *y* < 0.04 mm. Thus, the boundaries of the light zones and the dark zone were set as lines, where *y* = 0 mm. According to Yang et al. [34] and Ye et al. [8], the time during which the algal cells pass through the light/dark interface twice can be defined as a light/dark cycle period (*T*) consisting of light time (*t*_*l*_) and dark time (*t*_*d*_), and the average light/dark cycle period of the whole population can be calculated via

(10) $$\begin{array}{c} T=\underset{n\to \infty }{\lim}\left(\frac{1}{n}\cdot \overset{n}{\underset{i=1}{\sum }}\left(t_{l}+t_{d}\right)\right). \end{array}$$

The light/dark cycle frequency (*f*) and the light-time ratio (*φ*) were calculated using

(11) $$\begin{array}{c} f=\frac{1}{T}, \end{array}$$

(12) $$\begin{array}{c} \varphi =\underset{n\to \infty }{\lim}\left(\frac{1}{n}\cdot \overset{n}{\underset{i=1}{\sum }}\left(\frac{t_{l}}{t_{l}+t_{d}}\right)\right). \end{array}$$

## 3. Results and Discussion

### 3.1. Effect of the Aeration Direction on the Performance of the Novel PBRs

**Figure 2:**
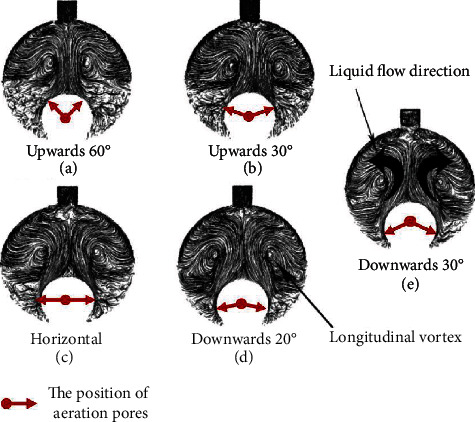
Flow fields of the novel tangent double-tube under different aeration directions at *z* = 500 mm.

In order to optimize the aeration direction, the flow field of the tangent double-tube PBR was simulated, and the values of the radial velocity (*V*_*r*_) and ∣cos*α*∣ were calculated. Based on an aeration rate of 0.3 vvm, the cross-sectional flow field characteristics at five different aeration directions (*z* = 500 mm) were analyzed. The path lines of different aeration directions in the cross-section of *z* = 500 mm are shown in Figures 2(a)–2(e), and the longitudinal vortices were generated. Compared to the five parts in Figures 2(a)–2(e), for the structure with upwards and horizontal aeration directions, the flow in the dark area is sparse and disordered, resulting in a poor mixing effect in the dark area. The poor mixing effect in the dark area not only hinders the complete dissolution and mixing of carbon dioxide and nutrients but also causes cell sedimentation [1].

However, in the downwards direction, the flow lines in the dark area are inerratic. Wu et al. [14] proposed using spiral tube PBRs, which can produce strong swirling motions near the wall; ultimately, no vortices were formed in the middle sections of these spiral tube PBRs. Compared to the spiral tube PBRs, the longitudinal vortices produced by the tangent double tube were clearer, especially for the downwards aerations. This phenomenon possible occurs because the main stream flows in the axial direction, and the spiral structure has little effect on the radial disturbance.

In addition, the vortices with greater energy and velocity drive the microalgae to shuttle continuously between the light and dark zones, which is beneficial for the photosynthesis of microalgae [1]. The analysis of photocatalytic oxidation reactors in the plate type showed that the generation of multiple longitudinal vortex flows effectively enhanced the mass transfer [24]. Thus, downwards aeration offers more opportunities for microalgae to obtain sufficient mixing in the dark area and was conducive to improving the mass transfer performance and flashing-light effect of the PBRs, thereby creating favorable conditions for the growth of microalgae.

**Figure 3:**
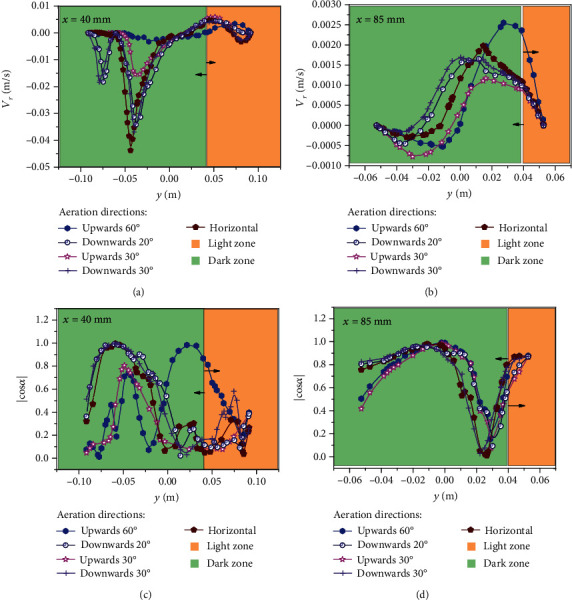
The *V*_*r*_ and ∣cos*α*∣ curves of the line *x* = 40 mm (a, c) and 85 mm (b, d) at aeration flow rates of 0.3 vvm under different aeration directions: upwards 60°, upwards 30°, horizontal, downwards 20°, and downwards 30°.

The microalgal growth rate was directly related to the velocity in the PBRs, which determined the mixing performance of the PBRs [35]. Figures 3(a)–3(d) show the relationship between *V*_*r*_ and ∣cos*α*∣ at *x* = 40 mm (around the aeration pores)/85 mm (around the wall) under different aeration directions. In Figure 3(a), for the upwards and horizontal aerations, the *V*_*r*_ value of the area below the tangent point of the double tube at the *x* = 40 mm line is close to 0 m/s, which indicates that the microalgae cells are likely to experience cell sedimentation here. Creswell note that when the velocity is around 0 m/s, microalgae will experience sedimentation, so the dead zone should be avoided as much as possible (*V*_*r*_ = 0 m/s) [6]. At the same time, for the new PBRs with downwards aeration, the velocity gradient distribution was large, and two peaks appeared in the dark area. This phenomenon indicates that downwards aeration can significantly improve the mixing effect. Wang et al. [32] found that the presence of a velocity gradient between the dark and light areas could allow algal cells to more effectively swim between the dark and light areas. For the novel PBRs proposed in this study, the streamline curves of all aeration directions in the light area are similar and have small values. Further, as can be seen in Figure 2, the flow area in the light area may be larger than that in the dark area. As shown in Figure 3(b), the value of *V*_*r*_ at the line of *x* = 85 mm is one order of magnitude smaller than the value of *V*_*r*_ at the line of *x* = 40 mm. This phenomenon may be due to anchorage dependence, similar to that reported by Haut et al. [36]. For all aeration directions, the enhancement of mass transfer near the wall of the PBRs was very weak, so attention should be paid to the periphery of the wall. In the area of *y* < 0.01 m, the *V*_*r*_ value of the PBR with downwards aeration of 30° was greater than that of the other PBRs. That is to say, aeration oriented 30° downwards achieved flow disturbance in the dark area, thereby reducing the mass boundary layer. In the area of *y* > 0.01 m, the PBR with aeration 60° upwards had the largest *V*_*r*_ value, making it the most suitable for mixing in the light area. However, in the dark area with aeration oriented upwards at 60°, the *V*_*r*_ value was lower. In addition, Gris et al. [37] showed that mixing along the light direction can improve light reception and mass transfer and produce a flashing-light effect, which may be beneficial for microalgal cultivation.

**Figure 4:**
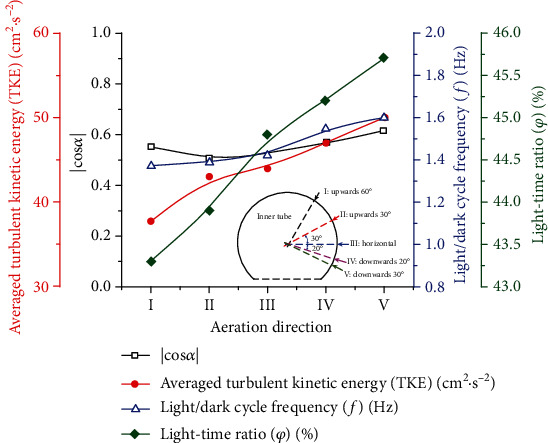
The average ∣cos*α*∣, average turbulent kinetic energy (TKE), light/dark cycle frequency (*f*), and light-time ratio (*φ*) at aeration flow rates of 0.3 vvm under different aeration directions.

According to Equations (1) and (2), the angle between the velocity field and the light direction represents the degree of coordination. As shown in Figure 3(c), in the area below *y* = 0 m, the ∣cos*α*∣ value of the upwardly aerated PBRs is similar. However, for the ∣cos*α*∣ at *y* = 0 m, the PBR with 60° upwards aeration has a higher ∣cos*α*∣ value than the PBR with 30° upwards aeration. Under horizontal and downwards aeration conditions, the peak of ∣cos*α*∣ is close to 1 in the dark area, which is greater than the peak value under upwards aeration conditions. However, in the light zone, the value of the ∣cos*α*∣ of the PBR with 60° upwards aeration is the largest. By comparing the average ∣cos*α*∣ values in the PBRs of all aeration directions, the following relationship is obtained: |cos*α*|_upwards 30°_ < |cos*α*|_upwards 60°_ < |cos*α*|_horizontal_ < |cos*α*|_downwards 20°_ < |cos*α*|_downwards 30°_. Therefore, the average ∣cos*α*∣ value of 0.521 was the largest under 30° downwards aeration. In Figure 3(d), at *x* = 85 mm, the ∣cos*α*∣ values of the PBRs with aeration structures of 20° and 30° downwards are greater than the ∣cos*α*∣ values of the other PBRs. At the same time, as shown in Figure 4, under 30° downwards aeration, the average value of ∣cos*α*∣ at *x* = 85 mm is the largest and reached 0.711. The maximum value is reached when the structure is aerated 30° downwards (0.616).

The TKE value reflects the mixing performance of a PBR. Many studies have shown that the higher the TKE is, the better the mixing performance of the PBR [10, 38, 39]. Figure 4 shows a relationship curve of the averaged turbulent kinetic energy (TKE) in different aeration directions. In Figure 4, the TKE of 30° downwards aeration is the greatest, increasing by approximately 35% to 50 cm^2^·s^−2^ compared to the TKE of 60° upwards aeration [10]. A higher TKE value indicates more effective mixing of the mass, which is associated with a more suitable environmental for microalgal growth [35]. In Figure 2, it can be seen that the inner tube with aeration can form multiple longitudinal vortices in the PBRs. The 30° downwards aeration structure can achieve a notably better mixing process, thereby greatly increasing the radial velocity of the microalgal solution and the TKE value.

In addition, as shown in Figure 4, when the aeration rate is 0.3 vvm, the light/dark cycle frequency and light-time ratio increase as the aeration direction rotates clockwise from top to bottom. The maximum light/dark cycle frequency and time ratios are achieved at 1.6 Hz and 46%, respectively, under the structure with 30° downwards aeration. The minimum light/dark cycle frequency and light-time ratio are achieved at 1.45 Hz and 44%, respectively, under the structure with 60° upwards aeration. In Figure 2, the structure with 60° upwards aeration provides less disturbance to the radial flow of microalgae solution than the structure with 30° downwards aeration. The structure with upwards aeration produces cell sedimentation in the lower dead zone, which results in disordered flow in the dark zone and insufficient radial velocity to push the microalgae cells back to the light zone. Meanwhile, the structure with upwards aeration produces a current in the longitudinal vortices of the light area. Although this phenomenon can increase the time the algal cells stay in the light area, it does not improve the flashing-light effect. According to previous research [8], increasing the flashing-light effect can effectively increase the photosynthesis of the algal cells. The structure with 30° downwards aeration enabled the longitudinal vortices to be located at the boundary between the light and dark zones, thus providing sufficient kinetic energy for the algal cells to swim between the light and dark zones. These phenomena provide a reasonable explanation for the optimal light/dark cycle frequency and the optimal light-time ratio between the light and dark areas caused by the 30° downwards aeration structure shown in Figure 4. In other words, adjusting the direction of the inner tube aeration can enhance the flashing-light effect of microalgae cells and can enhance the dissolution and diffusion of CO_2_ and improve the mixing of the nutrients. According to the above comparisons, the performance of the PBR can be comprehensively evaluated based on the average ∣cos*α*∣, the average turbulent kinetic energy (TKE), the light/dark cycle frequency (*f*), and the light-time ratio (*φ*). The 30° downwards aeration structure optimized the synergy and achieved the greatest mixing along the light direction, thereby improving the flashing-light effect and mass transfer and helping create the best microenvironment for algal cells.

**Figure 5:**
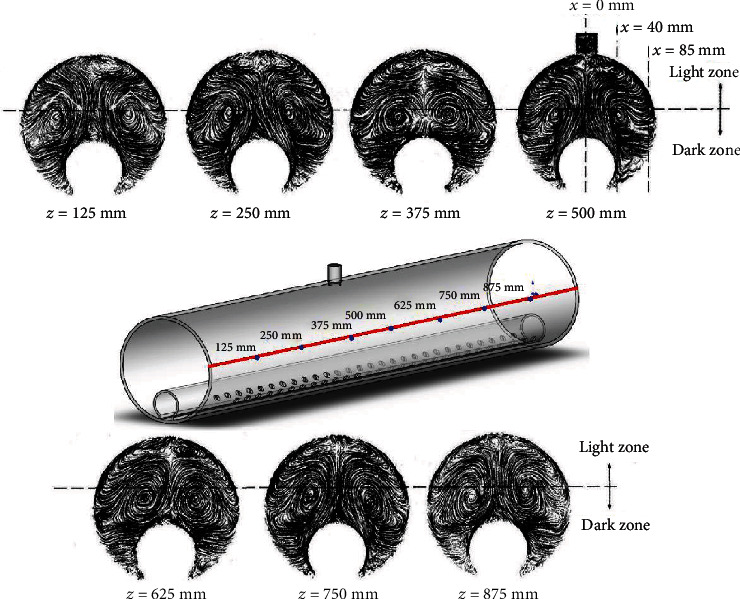
The flow characteristics of each section in the axial direction of the novel PBR under 30° downwards aeration.

Figure 5 presents the flow characteristics of each section in the axial direction of the novel PBR based on CFD. When the downwards aeration direction is 30°, longitudinal vortices are generated in each section of the PBR, and small vortices are present in the light zone. The main longitudinal vortices, with high energy and velocity, not only prevent the microalgae from sinking but also push the microalgae cells between the light and dark areas. In addition, small vortices in the light region at the top of the PBR facilitate full dissolution and gas- (CO_2_-) liquid (nutrient) mixing. Compared with the work of Cheng et al. [1] on plate PBRs, the main vortices generated here by the jet flow in the plate PBRs are beneficial to gas-liquid mixing, while the secondary vortices push the microalgae between the light and dark regions. However, these vortices have similar functions that improve mass transfer performance and the flashing-light effect of the PBR and promote the growth of microalgae. Taking the cross-section of *z* = 250 mm as an example, the vortex structure is symmetrically distributed on the left and right sides, and the streamline distribution in the dark zone shows that the gas-liquid two-phase flow significantly improved the mixing degree of the dark zone and was conducive to the swimming of algal cells toward the light zone. With constant CO_2_ gas in the outer tube, the energy of the main vortex is constantly replenished to maintain and reciprocate the vortex motion. Although some sections at 125 mm, 375 mm, 625 mm, and 875 mm do not have aeration pores, longitudinal vortices are still formed due to the flow around the surrounding aeration.

### 3.2. Effect of Aeration Rates on the Performance of the Novel PBRs

**Figure 6:**
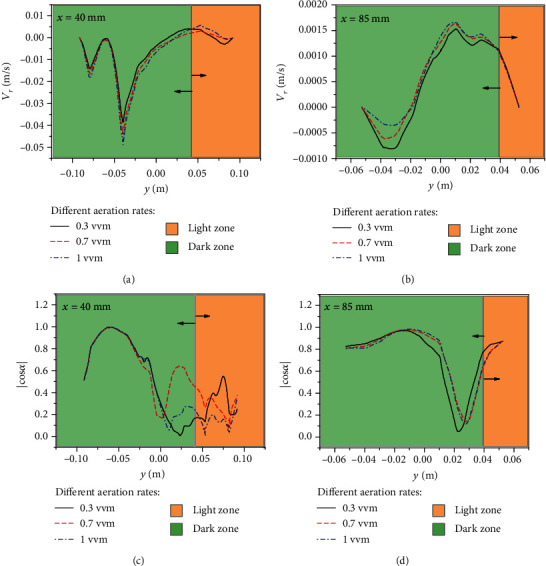
The *V*_*r*_ and ∣cos*α*∣ curves of the lines *x* = 40 mm (a, c) and 85 mm (b, d) at aeration flow rates of 0.3, 0.7, and 1.0 vvm based on 30° downwards aeration.

According to the above results, 30° downwards aeration was most beneficial to optimize the ∣cos*α*∣ and achieve higher mixing in the dark zones. Next, based on 30° downwards aeration, three aeration rates of 0.3 vvm, 0.7 vvm, and 1 vvm were compared to obtain the optimal aeration rate.  Figure 6 shows the curves of *V*_*r*_ and ∣cos*α*∣ for *x* = 40 mm and *x* = 85 mm under different aeration rates.

In Figure 6(a), the *V*_*r*_ at *x* = 40 mm slightly increases as the aeration rate increases. The peaks of *V*_*r*_ for 0.3 vvm, 0.7 vvm, and 1 vvm are 0.0393 m/s, 0.045 m/s, and 0.049 m/s, respectively. As shown in Figure 6(b), the *V*_*r*_ at *x* = 85 mm under the three aeration rates is one order smaller than that at *x* = 40 mm. Meanwhile, the *V*_*r*_ increases as the aeration rate increases at *y* < −0.01 m, while in other areas, the *V*_*r*_ value remains almost the same. As shown in Figure 6(c), at *x* = 40 mm, the three curves of ∣cos*α*∣ almost coincide, and the peak value is able to reach 0.996 at the bottom of the PBR. At the top of the dark area (0 < *y* < 0.04 m), under an aeration rate of 0.7 vvm, the synergistic effect is higher than that under other aeration rates and reaches a peak of 0.646. In the light zones, the ∣cos*α*∣ values are low. The value of ∣cos*α*∣ under 0.3 vvm is the largest, while the value of ∣cos*α*∣ under 1 vvm is the smallest. Meanwhile, the average ∣cos*α*∣ value is the largest (0.603) at 0.7 vvm. In Figure 6(d), in the dark zones at *y* < 0.03 m, all the values of ∣cos*α*∣ are close to 1. This gives the following relation: |cos*α*|_0.3_ < |cos*α*|_1.0_ ≈ |cos*α*|_0.7_.

**Figure 7:**
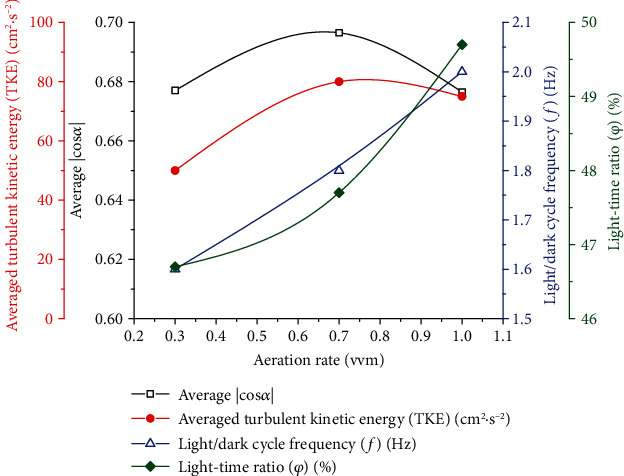
The average ∣cos*α*∣, average turbulent kinetic energy (TKE), light/dark cycle frequency (*f*), and light-time ratio (*φ*) at different aeration flow rates of 0.3 vvm, 0.7 vvm, and 1 vvm based on 30° downwards aeration.

In Figure 6, the maximum value of the cross-sectional average ∣cos*α*∣ is the largest under 0.7 vvm. Finally, as shown in Figure 7, an aeration rate of 0.7 vvm is more conducive to synergy between the velocity field and the direction of light. The radial velocities at different aeration rates are not significantly different. The average ∣cos*α*∣ value for 0.7 vvm (|cos*α*|_0.7_) is 0.69, which means that an aeration rate of 0.7 vvm is more conducive to synergy, as well as most conducive to mass transfer and light reception.

Figure 7 shows the effect of different aeration rates on the light/dark cycle frequency and light-time ratio of microalgae cells under the structure with downwards aeration of 30°. In algal cultivation, the flashing-light effect reflects the periodic exposure of algae to light and dark areas or rapid movement between light and dark areas. At an aeration rate of 0.3 vvm, the minimum light/dark cycle frequency and light-time rate were obtained as 1.65 Hz and 47.1%, respectively. When the aeration rate was 1.0 vvm, the maximum light/dark cycle frequency and light-time rate were 2.1 Hz and 49.1%, respectively. The structure with 30° downwards aeration enables the longitudinal vortices to be located at the boundary between the light and dark zones, thereby providing sufficient kinetic energy for algal cells to swim between the light and dark zones. When the aeration rate increased from 0.3 to 1.0 vvm, sufficient kinetic energy was given to the fluid; moreover, the presence of vortices and the enhancement of radial flow improved the mixing of the cell between the light and dark zones, thereby shortening the L/D cycle of microalgae cells. In addition, as shown in Figure 7, as the aeration volume increases, the TKE of the tangential PBR achieves its maximum value. When the aeration rate increases from 0.7 to 1.0 vvm, the value of TKE increases from 51 to 80 cm^2^·s^−2^. The vortex was ultimately the main disturbing force in the PBR. With an increase in the aeration rate, more kinetic energy was injected into the culture solution, which promoted the formation of a vortex [35, 39]. However, when the aeration rate was too high, the existence of a large number of bubbles prevented mixing of the gas-liquid phase to some extent. This phenomenon is similar to the study of jet aerated PBR by Cheng et al. [1]. Thus, combined with the degree of synergy, excessive gas content in the medium is not always conducive to the mixing of the solution along the light direction [8]. Therefore, when the aeration rate increased from 0.7 to 1.0 vvm, the values of TKE and ∣cos*α*∣ decreased by 4% and 6%, respectively. That is, when the aeration rate was 0.7 vvm, the TKE value was higher, and the mixing performance of the PBR was better. In short, although the light/dark cycle frequency (*f*) and the light-time ratio (*φ*) were best when the aeration rate was 1 vvm, the mixing performance was inferior to that at 0.7 vvm. Moreover, when the aeration rate was 0.7 vvm, the flashing-light effect was already satisfactory. Therefore, an aeration rate of 0.7 vvm is an effective choice for this novel PBR.

According to the above comparisons, the performance of the PBR can be comprehensively evaluated based on the average ∣cos*α*∣, average turbulent kinetic energy (TKE), light/dark cycle frequency (*f*), and light-time ratio (*φ*). A downwards aeration direction of 30° and an aeration rate of 0.7 vvm were the most conducive to reducing the dead zone and improving the light/dark cycle frequency, which improved the flashing-light effect and mass transfer and helped create a more suitable microenvironment for algal cells.

### 3.3. Performance Comparison of Tangent Double-Tube PBRs with Concentric Double-Tube PBRs

**Figure 8:**
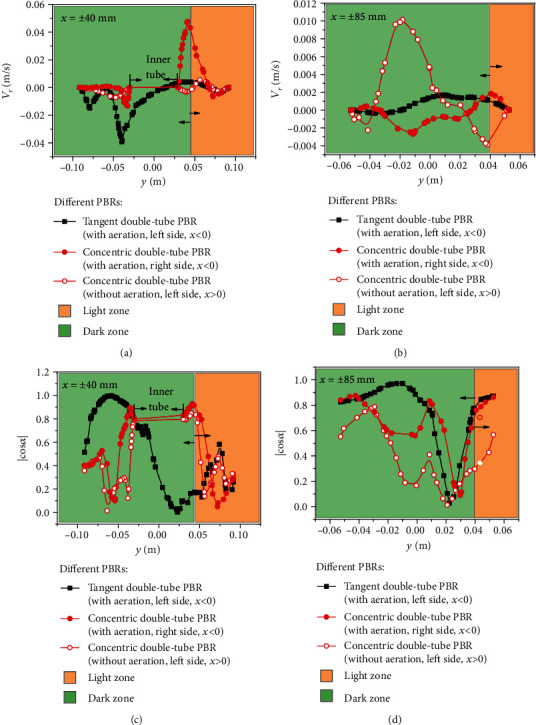
The *V*_*r*_ and ∣cos*α*∣ curves of the lines *x* = ±40 mm and *x* = ±85 mm in the tangent and concentric double-tube PBRs.

To better understand the advantages of tangent double-tube PBRs in this work, These PBRs were compared with the simulation result of the concentric double-tube PBR presented in Figure 1(a). For the concentric double-tube PBRs, the inner and outer tubes are coaxial, and aeration is accomplished via one row of pores located on the inner-tube [25]. The flow in the tangent double-tube PBR is symmetrical. However, the flow field is distributed differently on both sides of the concentric PBR because the aeration is located on only one side (*x* < 0). Therefore, during the analysis, four lines of *x* = ±40 mm and *x* = ±85 mm from the concentric PBR were selected. The curves of *V*_*r*_ and ∣cos*α*∣ in the two PBRs are presented in Figures 8(a)–8(d).

As shown in Figure 8(a), in the dark area, the *V*_*r*_ value of the tangent double-tube PBR is higher with two peak velocities, while at *x* = 40 mm, the *V*_*r*_ value of the concentric double-tube PBR is very low. However, in the light zones of *x* = −40 mm, a peak appeared in the *V*_*r*_ of the concentric double-tube PBR, reaching its maximum (0.047 m·s^−1^). These data demonstrate that the comprehensive mixing effect was better in the tangent double-tube PBR. As shown in Figure 8(b), the *V*_*r*_ for the line at *x* = 85 mm in the two PBRs was one order lower than that for the line at *x* = 40 mm. However, the *V*_*r*_ of the concentric structure was larger near the wall, which indicates that enhancing the flow disturbance near the wall is an important consideration for improving the tangent double-tube PBR. This phenomenon seems to be due to the dispersion of power caused by the aeration on both sides. A comparison of the radial velocity showed that the tangent double-tube PBR offers better mixing performance, but the tangent double-tube PBR provides weaker interference on the boundary layer than the concentric double-tube PBR.

As shown in Figure 8(c), in the dark zones of *x* = ±40 mm, the ∣cos*α*∣ value of the tangent double-tube PBR is larger than that of the concentric double-tube PBR, which shows that the synergy of the tangent double-tube PBR was better in the dark zones. However, in the light zones of 0.04 m < *y* < 0.06 m, the ∣cos*α*∣ value of the concentric double-tube PBR is higher, while in the light zones of *y* > 0.06 m, the ∣cos*α*∣ of the concentric double-tube PBR is lower. This phenomenon might be due to wall interference from the inner tube. In the light zones of 0.04 m < *y* < 0.06 m, the flow field of the concentric double-tube PBR was disturbed by the inner tube wall surface, and the fluid velocity near the boundary layer was reduced to make it suitable for the growth of microalgae. Compared to the tangent double-tube PBR at the same position, the flow velocity of the tangent double-tube PBR at 0.04 m < *y* < 0.06 m is higher than that of the concentric PBR due to the lack of inner wall interference. Therefore, the ∣cos*α*∣ value of the concentric double-tube PBR was temporarily higher than the ∣cos*α*∣ value of the tangent double-tube PBR. When the fluid in the concentric PBR was far away from the surface of the inner tube wall, the boundary layer effect disappeared, as the excessive fluid velocity was no longer a component suitable for the environment. Moreover, the ∣cos*α*∣ value of the concentric double-tube PBR decreased.

As shown in Figure 8(d), on the line at *x* = ±85 mm, in most areas, the ∣cos*α*∣ value of the tangent double-tube PBR is greater than the ∣cos*α*∣ value of the concentric double-tube PBR. For the range of 0.005 m < *y* < 0.028 m on the line at *x* = −85 mm, the ∣cos*α*∣ value of the concentric double-tube PBR was slightly larger. In Table 1, the average ∣cos*α*∣ value of the tangent double-tube PBR is 0.649, which is greater than the average ∣cos*α*∣ value (0.508) of the concentric double-tube PBR at 0.3 vvm. Therefore, the synergy extent of the tangent double-tube PBR was better and more beneficial to mass transfer. Through the comparisons of *V*_*r*_ and ∣cos*α*∣ above, the flow disturbance of the dark zones in the tangent double-tube PBR and the synergy between the light direction and velocity field were both shown to be better, but the flow disturbance near the wall is the key position for optimizing the tangential double-tube PBR structure.

In this work, concentric double-tube PBRs and tangent double-tube PBRs were used as the simulation objects, and the flow characteristics and flashing-light effect of the tangent double-tube PBRs and the concentric double-tube PBRs were investigated using the hydrodynamics method. It is well known that a variety of tube PBRs have been designed for microalgae cultures [1] and that experimental characterization of the flow field in a PBR is difficult and expensive [8]. Therefore, computational fluid dynamics (CFD) is a significant, popular, and reliable method for measuring the internal performance of a tube PBR [16].

In our previous work [25], a concentric double-tube structure was manufactured and studied experimentally in the laboratory. The potential of use of a concentric double-tube PBR for microalgae cultures was verified through microalgae culture experiments. In particular, the concentric double-tube PBR was more amenable to the growth of microalgae. The experimental data showed that the biomass yield of the concentric double-tube PBR increased by at least 43.6% to 107.4% compared to the common tubular PBR. At the same time, field synergy theory combined with the concentric double-tube PBR simulation mode was applied to the design of tubular microalgae PBRs. The light and velocity gradients for the cross-section of the synergy degree were higher on average (∣cos*α* | = 0.5) for the concentric double-tube PBR experimental results (which provides a theoretical explanation), and the simulation results show that, for PBRs, a TKE value up to 54 cm^2^·s^−2^ is superior to other values. During the 15-day culture process, the pH value of the concentric double-tube PBR changed from 7.5 to 9.0, while that of the common PBR changed from 7.5 to 8.8. The dissolved oxygen concentration of the PBR fluctuated between 6.0 and 7.0 mg·L^−1^, while that of the common PBR fluctuated between 6.6 and 10.2 mg·L^−1^. These experimental and simulated data show that the mixing performance of the concentric double-tube PBR was obviously better than that of the common PBR. In other words, the experimental results verify the reliability of the two-tube model. In this paper, the simulated models of the tangent double-tube PBR were developed based on a concentric double-tube PBR model and a physical model with high similarity. The simulation results show that, compared to the concentric double-tube PBR, the light/dark cycle frequency and light time of the tangent double-tube PBR increased by 78.2% and 36.2% to 1.8 Hz and 47.8%, respectively, and the TKE was enhanced by 48.1%, from 54 to 80 cm^2^·s^−2^. The average synergy of the light and velocity gradients on the cross-section increased by 38% to 0.69. In general, the models of the tangent double-tube PBR and the concentric double-tube PBR established in this work are reliable, and the tangent double-tube PBR has greater potential than the concentric double-tube PBR. In other words, the tangent double-tube PBR is a more attractive and functional choice for microalgae cultures.

### 3.4. Performance Comparison with Other Tubular PBRs

As shown in Table 1, we compared the PBR proposed in this paper to other novel tubular PBRs proposed in recent studies. The TKE value is a key parameter for the mixing performance of a PBR. Compared to the concentric double-tube PBR, the TKE value of the tangent double-tube PBR increased by nearly 48.1%. Xu et al. [38] proposed a draft-tube airlift PBR for *Botryococcus braunii*, whose TKE value increased from 32 to 74 cm^2^·s^−2^ by optimizing its internal structure. Azizi et al. [10] also found that after a static mixer was embedded in the tubular PBR, the mixing conditions and hydrodynamic performance in the PBR were better than those of a traditional PBR, and the TKE value was able to reach 23 cm^2^·s^−2^, which is basically consistent with the tubular PBR research results of Ye et al. [8] using a four-cell Kenics mixer and a one-cell Kenics mixer (TKE = 26.6 cm^2^·s^−2^). Moreover, these studies were mainly designed to adjust the physical locations to improve the mixing performance of a culture solution. The novel PBRs proposed in this work adopted a tangential inner tube aeration structure, which generated symmetrical vertical vortices between the light and dark zones of the PBR. These novel structures promoted the mixing performance of the solution and significantly increased the TKE value (up to 80 cm^2^·s^−2^). The research focus of this study was similar to that of Cheng et al. [1], who improved mixing performance by using jet flow in a plate PBR, increasing the TKE by about 35.5%. In this work, compared to adding a static mixer or a draft tube, using an adjustable aeration method more effectively improved the mixing performance (or TKE value) of the culture medium. In other words, the tangent double-tube PBR offered better mixing performance and was more suitable for microalgae growth.

As shown in Table 1, the performance of the tangent double-tube PBR is superior to that of the concentric double-tube PBR in terms of its light/dark cycle frequency and light-time rate. In particular, the *f* of the tangent tube PBR is 1.8 times higher than that of the concentric double-tube PBR. This is mainly because the tangent double-tube PBR generated vortices between the light zone and the dark zone, which reduced the dead zone of the PBR and allowed the microalgae cells to effectively swim between the light zone and the dark zone. According to previous studies, the light/dark cycle frequency and light-time rate of tubular PBR have been widely explored [8, 13, 16]. Ye et al. [8] proposed a serial lantern-shaped draft tube PBR that could effectively improve the flashing-light effect of PBRs. Similar to the study by Azizi et al. [10], adding static mixers, such as Kenics mixer units, can also improve the mixing performance of a PBR. However, these methods did not generate obvious vortices in the culture medium, and improvement of the light/dark cycle frequency and light-time rate was limited. Gómez-Pérez et al. [16] developed a spiral PBR that could generate an obvious eddy current in the culture solution, which significantly improved the light/dark cycle frequency of the PBR, reaching as high as 0.9 Hz, similar to the concentric double-tube PBR. Next, Lei et al. [13] designed a novel tubular PBR with spiral ribs with a maximum light-dark cycle of 1.4 Hz, which is higher than that proposed by Gómez-Pérez et al. [16] and the concentric double-tube PBR but lower than the tangent double-tube PBR (1.8 Hz). According to Cheng et al. [1, 8], a suitable velocity flow field and mixing ability can provide an appropriate growth microenvironment for microalgae. Uniform velocity distribution can facilitate the uniform distribution of microalgae in the PBR, thereby minimizing aggregation or precipitation and promoting the penetration and uniform distribution of light, which will promote the growth of microalgae. At the same time, the complex vortex flow field in the tangential double-tube PBR, especially the velocity component along the light penetration direction, caused the microalgae to move back and forth between the light and dark zone solutions, an activity more conducive to photosynthesis (see Figure 2).

According to our previous studies [25], the concentric double-tube PBR formed large dead zones in the dark zone, causing the microalgae cells to remain in the dark zone for a long period of time. Therefore, in a concentric double-tube PBR, it is difficult for these microalgae cells to swim upwards to obtain light. Consequently, the light/dark cycle frequency and light-time ratio of the tangent double-tube PBR were much higher than those of the concentric double-tube PBR. The existing experiments showed that under some cultivation conditions, the volume production rate of *Chlorella vulgaris* in the concentric double tube increased by 137.50% compared to that in the tubular PBR [25]. When the aeration direction was located 30° downwards and the aeration rate was 0.7 vvm, compared to the concentric double-tube PBR, the tangent double-tube PBR of the light/dark cycle frequency and light-time ratio increased by 78.2% and 36.2% to 1.8 Hz and 47.8%, respectively. The cross-section of the synergy with the mean light and the velocity gradient increased by 38% to 0.69. In general, the tangent double-tube PBR was more beneficial to enhancing the mass transfer and improving the photosynthetic efficiency of algal cells; it will thus have greater potential for large-scale cultivation in a culture experiment. In our follow-up research, we will build an experimental model of a tangential double-tube PBR at laboratory scale and further discuss its performance. The simulation work in this paper provides theoretical justification for future experiments.

## 4. Conclusions

In this study, a novel tangent double-tube PBR with radial aeration pores along the tube length was proposed based on field synergy theory and simulated using CFD simulation technology. The simulation results are as follows:

The tangential inner tube aeration structure generated symmetrical vertical vortices between the light and dark areas in the PBR, and the novel PBR significantly enhanced the mass transfer, strengthened the flashing-light effect, and reduced the dead zone. Compared to the concentric double-tube PBR, the light/dark cycle frequency and light-time ratio of the tangent double-tube PBR increased by 78.2% and 36.2% to 1.8 Hz and 47.8%, respectively

A downwards aeration direction of 30° and an aeration rate of 0.7 vvm were the most conducive to enhancing the mixing performance of the novel PBR, and the average turbulent kinetic energy (TKE) was enhanced by 48.1%, from 54 to 80 cm^2^·s^−2^

Field synergy theory can be applied to the design of tubular microalgae PBRs. Compared to the concentric double-tube PBR, the average synergy of the light and velocity gradients across the cross-section increased by 38% to 0.69 in the tangent double-tube PBR. This novel design can provide a more suitable microenvironment for microalgal cultivation and holds promise for bioresource recovery and improving the yield of microalgae

## Figures and Tables

**Table 1:** Comparison of performance of the different tubular photobioreactors (PBRs).

PBR characteristics	∣cos*α*∣	TKE (cm^2^·s^−2^)	*f* (Hz)	*φ* (%)	Reference
Tangent double-tube PBRs	0.69	80	1.8	47. 8	This work
Concentric double-tube PBRs	0.5	54	1.01	35.1	This work
Serial lantern-shaped draft tube PBRs	—	26.6	0.476	32.9	[8]
Novel static mixers inside the tubular PBR	—	23	—	—	[10]
Successive and independent arrangement of Kenics mixer units in tubular PBRs	—	—	0.59-0.64	—	[11]
Tubular PBRs with spiral ribs	—	—	1.0-1.4 Hz	—	[13]
Twisted tubular photobioreactor	—	—	0.9-4.0	—	[16]
A draft-tube airlift PBR for *Botryococcus braunii*	—	32-74	0.7 ± 0.02	—	[38]

“—” means “not calculated in the literature”.

## Data Availability

The analysis data used to support the findings of this study are included within the article.

## Nomenclature

### Abbreviations

L/D: Light and dark cycle period
TKE: Turbulent kinetic energy (cm^2^·s^−2^)
PBR: Photobioreactor.

### Subscripts

*k*: The turbulence energy item
*q*: The *q* phase
*T*: The turbulent term
*ε*: Turbulent dissipation item.

### Symbols

*C*: Model constants
*d*: The diameter of solid particle (*μ*m)
$\overset{⃑}{F}$: The body force (kg·m^−2^·s^−2^)
*F*_*P*_: Pressure gradient force (kg·m^−2^·s^−2^)
*G*: The turbulence energy resulted from the average velocity gradient (cm^2^·s^−2^)
*p*: Partial pressure (Pa)
*t*: Time (s)
*T*: Light/dark cycle time (s)
$\vec{U}$: The flow velocity vector (m·s^−1^)
${\vec{v}}_{d}$: The particle velocity vector (m·s^−1^)
*ρ*_*q*_: The density of liquid (kg·m^−3^)
*r*: Volume fraction
*μ*_*t*_: The turbulence viscosity coefficient (kg·m^−2^·s^−2^)
*σ*: The turbulent Prandtl numbers
*ρ*_*d*_: The density of solid particle (kg·m^**−**3^)
*V*_*r*_: the radial velocity of fluid (m·s^−1^)
*C*_*D*_: A drag coefficient required to compute the drag force
*f*: The light/dark cycle frequency (Hz)
$\overset{⃑}{F_{lift}}$: The lift force (kg·m^−2^·s^−2^)
*F*_*D*_: Drag force (kg·m^−2^·s^−2^)
*n*: Algal cells with a number
*t*_*l*_: Light time (s)
*t*_*d*_: Dark time (s)
∇**T**: Temperature gradient (K)
${\vec{v}}_{f}$: The velocity vector (m·s^−1^)
|cos*α*|: Synergy extent
*φ*: The light-time ratio
*ρ*: Density (kg·m^−3^)
*П*: The impact of dispersed phase on the continuous phase
*α*: The angle between the direction of flow velocity and the direction of heat flux.

## References

[1] Cheng J., Lai X., Ye Q., Guo W., Zhou J. (2020). Numerical simulation on optimizing flow field and flashing-light effect in jet-aerated tangential swirling-flow plate photobioreactor to improve microalgal growth. *Chemical Engineering Science*.

[2] Zhu C., Zhai X., Jia J. (2018). Seawater desalination concentrate for cultivation of _Dunaliella salina_ with floating photobioreactor to produce *β*-carotene. *Algal Research*.

[3] Zhu C., Zhang R., Cheng L., Chi Z. (2018). A recycling culture of *Neochloris oleoabundans* in a bicarbonate-based integrated carbon capture and algae production system with harvesting by auto-flocculation. *Biotechnology for Biofuels*.

[4] Valente A., Iribarren D., Dufour J. (2019). How do methodological choices affect the carbon footprint of microalgal biodiesel? A harmonised life cycle assessment. *Journal of Cleaner Production*.

[5] Sibi G., Shetty V., Mokashi K. (2016). Enhanced lipid productivity approaches in microalgae as an alternate for fossil fuels - a review. *Journal of the Energy Institute*.

[6] Creswell L. (2010). *Phytoplankton Culture for Aquaculture Feed*.

[7] Suparmaniam U., Lam M. K., Uemura Y., Lim J. W., Lee K. T., Shuit S. H. (2019). Insights into the microalgal cultivation technology and harvesting process for biofuel production: a review. *Renewable and Sustainable Energy Reviews*.

[8] Ye Q., Cheng J., Guo W., Xu J., Li H., Zhou J. (2018). Numerical simulation on promoting light/dark cycle frequency to improve microalgae growth in photobioreactor with serial lantern-shaped draft tube. *Bioresource Technology*.

[9] Liao Q., Li L., Chen R., Zhu X. (2014). A novel photobioreactor generating the light/dark cycle to improve microalgal cultivation. *Bioresource Technology*.

[10] Azizi K., Moraveji M. K., Hassanzadeh H., Najafabadi H. A. (2018). Consideration of inclined mixers embedded inside a photobioreactor for microalgal cultivation using computational fluid dynamic and particle image velocimetry measurement. *Journal of Cleaner Production*.

[11] Qin C., Wu J. (2019). Influence of successive and independent arrangement of Kenics mixer units on light/dark cycle and energy consumption in a tubular microalgae photobioreactor. *Algal Research*.

[12] Gómez-Pérez C. A., Espinosa J., Ruiz L. C. M., van Boxtel A. J. B. (2015). CFD simulation for reduced energy costs in tubular photobioreactors using wall turbulence promoters. *Algal Research*.

[13] Lei Y., Wang J., Wu J. (2019). Optimization of Tubular Microalgal Photobioreactors with Spiral Ribs under Single-Sided and Double-Sided Illuminations. *Processes*.

[14] Wu L. B., Li Z., Song Y. Z. (2009). Numerical investigation of flow characteristics and irradiance history in a novel photobioreactor. *African Journal of Biotechnology*.

[15] Perner-Nochta I., Posten C. (2007). Simulations of light intensity variation in photobioreactors. *Journal of Biotechnology*.

[16] Gómez-Pérez C. A., Oviedo J. J. E., Ruiz L. C. M., van Boxtel A. J. B. (2017). Twisted tubular photobioreactor fluid dynamics evaluation for energy consumption minimization. *Algal Research*.

[17] Su Z., Kang R., Shi S., Cong W., Cai Z. (2010). Study on the destabilization mixing in the flat plate photobioreactor by means of CFD. *Biomass and Bioenergy*.

[18] Pruvost J., Pottier L., Legrand J. (2006). Numerical investigation of hydrodynamic and mixing conditions in a torus photobioreactor. *Chemical Engineering Science*.

[19] Pruvost J., Legrand J., Legentilhomme P., Muller-Feuga A. (2004). Effect of Inlet Type on Shear Stress and Mixing in an Annular Photobioreactor Involving a Swirling Decaying Flow. *The Canadian Journal of Chemical Engineering*.

[20] Sato T., Usui S., Tsuchiya Y., Kondo Y. (2006). Invention of outdoor closed type photobioreactor for microalgae. *Energy Conversion and Management*.

[21] Guo Z. Y., Li D. Y., Wang B. X. (1998). A novel concept for convective heat transfer enhancement. *International Journal of Heat and Mass Transfer*.

[22] Liu W., Liu Z., Huang S. (2010). Physical quantity synergy in the field of turbulent heat transfer and its analysis for heat transfer enhancement. *Chinese Science Bulletin*.

[23] Wei L., Zhichun L., Tingzhen M., Zengyuan G. (2009). Physical quantity synergy in laminar flow field and its application in heat transfer enhancement. *International Journal of Heat and Mass Transfer*.

[24] Chen Q., Ren J., Guo Z. (2008). Field synergy analysis and optimization of decontamination ventilation designs. *International Journal of Heat and Mass Transfer*.

[25] Yang J., Cui X., Feng Y., Jing G., Kang L., Luo M. (2017). Experimental study on microalgal cultivation in novel photobioreactor of concentric double tubes with aeration pores along tube length direction. *International Journal of Green Energy*.

[26] Fernández F. A., Camacho F. G., Pérez J. S., Sevilla J. F., Grima E. M. (1997). A model for light distribution and average solar irradiance inside outdoor tubular photobioreactors for the microalgal mass culture. *Biotechnology and Bioengineering*.

[27] Barbosa M. J., Janssen M., Ham N., Tramper J., Wijffels R. H. (2003). Microalgal cultivation in air-lift reactors: modeling biomass yield and growth rate as a function of mixing frequency. *Biotechnology and Bioengineering*.

[28] Wileman A., Ozkan A., Berberoglu H. (2012). Rheological properties of algae slurries for minimizing harvesting energy requirements in biofuel production. *Bioresource Technology*.

[29] McHardy C., Luzi G., Lindenberger C., Agudo J. R., Delgado A., Rauh C. (2018). Numerical analysis of the effects of air on light distribution in a bubble column photobioreactor. *Algal Research*.

[30] Fu J., Huang Y., Liao Q., Xia A., Fu Q., Zhu X. (2019). Photo-bioreactor design for microalgae: a review from the aspect of CO_2_ transfer and conversion. *Bioresource Technology*.

[31] Saini R. K., Wangikar P. P., Bose M. (2018). CFD analysis of the flow dynamics of microorganisms in dilute cultures in stirred tank photobioreactors. *Bioresource Technology Reports*.

[32] Wang L., Tao Y., Mao X. (2014). A novel flat plate algal bioreactor with horizontal baffles: structural optimization and cultivation performance. *Bioresource Technology*.

[33] Luo H.-P., Al-Dahhan M. H. (2011). Verification and validation of CFD simulations for local flow dynamics in a draft tube airlift bioreactor. *Chemical Engineering Science*.

[34] Yang Z., Cheng J., Ye Q., Liu J., Zhou J., Cen K. (2016). Decrease in light/dark cycle of microalgal cells with computational fluid dynamics simulation to improve microalgal growth in a raceway pond. *Bioresource Technology*.

[35] Huang J., Li Y., Wan M. (2014). Novel flat-plate photobioreactors for microalgal cultivation with special mixers to promote mixing along the light gradient. *Bioresource Technology*.

[36] Haut B., Ben Amor H., Coulon L., Jacquet A., Halloin V. (2003). Hydrodynamics and mass transfer in a Couette-Taylor bioreactor for the culture of animal cells. *Chemical Engineering Science*.

[37] Gris B., Morosinotto T., Giacometti G. M., Bertucco A., Sforza E. (2014). Cultivation of *Scenedesmus obliquus* in photobioreactors: effects of light intensities and light-dark cycles on growth, productivity, and biochemical composition. *Applied Biochemistry and Biotechnology*.

[38] Xu L., Liu R., Wang F., Liu C. (2012). Development of a draft-tube airlift bioreactor for *Botryococcus braunii* with an optimized inner structure using computational fluid dynamics. *Bioresource Technology*.

[39] Zhang Q. H., Wu X., Xue S. Z., Wang Z. H., Yan C. H., Cong W. (2013). Hydrodynamic characteristics and microalgal cultivation in a novel flat-plate photobioreactor. *Biotechnology Progress*.

